# Regulatory axes on food advertising to children on television

**DOI:** 10.1186/1743-8462-6-1

**Published:** 2009-01-22

**Authors:** Elizabeth Handsley, Kaye Mehta, John Coveney, Chris Nehmy

**Affiliations:** 1 School of Law, Flinders University, Adelaide, Australia; 2 School of Nutrition and Dietetics, Flinders University, Adelaide, Australia; 3 School of Public Health, Flinders University, Adelaide, Australia

## Abstract

This article describes and evaluates some of the criteria on the basis of which food advertising to children on television could be regulated, including controls that revolve around the type of television programme, the type of product, the target audience and the time of day. Each of these criteria potentially functions as a conceptual device or "axis" around which regulation rotates. The article considers examples from a variety of jurisdictions around the world, including Sweden and Quebec. The article argues that restrictions centring on the time of day when a substantial proportion of children are expected to be watching television are likely to be the easiest for consumers to understand, and the most effective in limiting children's exposure to advertising.

## Background

When it comes to television food advertising and childhood obesity, it seems that everybody has an axe to grind. Health and consumer groups claim that food advertising contributes to an obesegenic environment for children, and should be curtailed, if not banned outright [1]. The broadcasting, advertising and food and beverage industries dispute that regulation can help curb the rate of childhood obesity, and (yet) insist they want to be part of the solution [2]. The television production industry is concerned that additional restrictions on advertising to children could cause the market in children's programs to dry up [3], and in Australia at least, the regulator appears to have accepted such arguments [4]. The previous Australian government refused to take action on the ground that children's diets and lifestyles are a matter of personal choice and parental responsibility [5], and during the 2007 election campaign Kevin Rudd, the new Prime Minister, more or less agreed [6]. As with many complex debates where the stakes are high, participants are often at cross-purposes. In particular, advocates of stricter regulation do not claim that food advertising is the only cause of childhood obesity, and yet they are frequently portrayed as espousing just such a view.

Recent studies have found a direct correlation between children's exposure to food advertisements and their food preferences [7,8]. Research also confirms that the majority of foods advertised on television during periods when children are likely to be watching are high in sugar and fat [9]. If preferences are a predictor of actual consumption, then it is reasonable to conclude that television advertising leads children to consume more food of low nutritional value – which is often calorie dense and contains high levels of sugar and fat – than they would otherwise do. On this view, children's daily energy intake is being inflated without corresponding increases in opportunities for physical activity. In public health parlance, food advertising to children contributes to an "obesegenic" environment.

At the time of writing there is debate in numerous countries as to *whether *food advertising should be more tightly regulated. If that debate is resolved in favour of tighter regulation – if the axe falls – the next question will be *how *best to regulate food advertising in order to reduce children's exposure to it. This article evaluates some of the core concepts through which regulators could differentiate between advertising that is subjected to lighter, and heavier, regulatory burdens. It is helpful to think of the conceptual device used in each case as an "axis" around which regulation rotates. This allows an appraisal of different forms of regulatory options, each turning on a certain element of the advertising or context in which it is shown.

This article identifies a selection of regulatory axes currently in use and evaluates their relative likely effectiveness in achieving the goals of usability by average viewers and of limiting children's exposure to advertising that could influence their food preferences. In the interests of brevity, this article deals only briefly with regulatory controls that focus on the content or effect of the advertisement; for example, advertisements that have the effect of placing pressure on children to request the product from their parents ("pester power"), or advertisements that contain inaccurate health or nutritional information. Information and references in the article are current to February 2008 (but the authors note in particular that a revised version of the AANA code on advertising to children was introduced in April 2008).

The first point to make is that regulatory axes need to provide criteria that are clear and easy to understand. This is particularly the case if the regulatory system relies on consumer complaints to activate its machinery, as is the case in Australia. In order to maximise the likelihood that consumers will recognise breaches and take the trouble to complain, regulations should use criteria that are meaningful to the average person. Such a person is not likely to bother complaining unless he or she can be reasonably confident that a breach has occurred.

Not all criteria being used around the world at the moment are consumer-friendly in this sense. Many are vague and inherently open to interpretation, or such that reasonable minds may differ as to whether they have been met. In law we may be accustomed to such standards; arguing over them is the stock in trade of lawyers. It is different, however, in consumer-protection regulation. Consumer complaints mechanisms do not support systematic input by professionals on both sides of the argument. Therefore the industry side tends to carry the day, even if a consumer does take the trouble to mount the argument that a regulatory standard has been breached.

Second, regulation needs to limit the opportunities for food advertising to influence children's food preferences, as this is the role that the research has identified for food advertising in contributing to childhood obesity. To borrow a term that has been used frequently by the High Court of Australia in determining the constitutionality of legislation, what is needed are criteria that are "appropriate and adapted" [10] to reducing children's exposure to food advertising, or to moderating the impact of food advertising on children's food preferences. As will be seen, not all criteria fit this description.

Although this article is not (only) about the Australian regulatory system for advertising on commercial free-to-air television, it may be useful to provide a brief description of its key elements. Australia has a system of co-regulation, meaning that both government and industry have a role. The government contributes through the Australian Communications and Media Authority (ACMA), which exercises a range of functions under the *Broadcasting Services Act 1992 *(Cth), including propounding and enforcing the Children's Television Standards (CTS). These impose content quotas on commercial free-to-air broadcasters relating to quality children's programming, and they also impose certain restrictions on the advertising that can be shown at the time when that programming is scheduled. The CTS rely on the public to notice breaches of the provisions and to complain to ACMA. A range of civil sanctions is available for breaches of the CTS. At the time of writing the CTS are under review.

The industry body Free TV Australia contributes by developing and maintaining the Commercial Television Industry Code of Practice (CTICP), which covers a range of matters listed in the *Broadcasting Services Act *and is registered by ACMA in accordance with that Act. Among other things the CTICP extends the CTS advertising restrictions to advertisements "directed to children". It also contains certain additional restrictions on advertising directed to children that are not found in the CTS. As with the CTS, enforcement relies on consumer complaints, but in this case the complaints must go first to the broadcaster. A consumer who is unsatisfied with the broadcaster's response to the complaint can then take the matter to ACMA.

The advertising industry has contributed to the regulatory environment by developing a number of codes (AANA Codes) that apply not just to television but to all media. These do not have any legislative basis and the government has no role in their enforcement. Complaints are determined by the Advertising Standards Board, an industry body. On the other hand, some of the AANA Codes have been incorporated into the CTICP.

This article now provides a selective review of those "regulatory axes" that currently apply, or could apply, to food advertising to children.

## The criteria

### 1. The type of program

Some regulations apply only to advertising shown during children's programs. There are a number of ways of defining this category.

#### Dedicated children's programming

In Australia, the regulations that are directly enforced by the government, the Children's Television Standards, apply during periods that licensees have set aside for "C" programs, that is, dedicated children's programming that has been classified as meeting certain regulatory criteria. These programs are broadcast in fulfilment of a quota imposed on commercial free to air broadcasters. Audiences for these programs are relatively small, therefore the advertising restrictions have little impact on the amount or kind of advertising to which children are exposed.

There are broader approaches to the idea of "children's programming" that do not involve a need for formal classification. For example section 7b of the Swedish *Radio and Television Act 1996 *requires the regulator to determine to whom the programme is addressed:

... programmes primarily addressed to children under twelve years of age may not be interrupted by advertising. ... Commercial advertising may not occur immediately before or after a programme or part of a programme that is primarily addressed to children under twelve years of age [with certain exceptions].

Similarly, the new British regulations apply to programs "specifically made for children" and programs "of particular appeal to children" [11].

Such approaches, however, necessarily introduce a measure of vagueness. For example, what does it mean to say a program is "of particular appeal to children"? Does it mean that the program appeals more to children than to adults? Or that it appeals to children more than other programs do? Clearly the reach and effectiveness of the regulation in limiting children's exposure to food advertising will vary depending on how this question is answered. And even once the question is answered it will not always be easy to categorise any given program.

Another problem with a regulatory axis that focuses on the type of program is that it tends to overlook the fact that children watch many shows that are not "of particular appeal" to them in either of the above senses. Young children might not be terribly attracted by a prime-time sitcom such as *How I Met Your Mother*, for example, but if their parents are watching it, the children probably will too. And even if they are not interested in the programme, chances are their attention will be drawn by the advertisements. Therefore they are still exposed to advertising, no less than if the programme were of primary interest to them, however that might be defined.

### 2. The type of product

#### Food generally

Food itself does not cause obesity; rather, obesity tends to be the result of certain patterns of consumption of food, and especially particular types of food. Indeed, food is necessary for life. Therefore, it would be politically unpalatable to introduce a regulation to do away with all advertising of any kind of food, as in principle, it would be taking matters too far.

However, it is difficult to distinguish between foods that should and should not be caught by an anti-obesity regulation, and it may lead to excessive hair-splitting and interpretive debate. Moreover, it is arguable that if food is necessary for life, it should not be necessary to advertise it! If a general ban on food advertising were applied, how much beneficial food advertising would be caught? According to the Australian lobby group, the Coalition on Food Advertising to Children, the answer is: not very much. Therefore it advocates a ban on *all *commercial food advertising during times when a substantial proportion of children are in the audience [1].

Since "food" as a category has meaning to the average consumer, it would normally be fairly easy to tell if a particular advertisement was caught by any regulation using it as an axis. The main reservation to this would relate to advertisements structured in such a way that they are for a company that sells food, rather than for a particular food product. Sometimes such companies have campaigns that do not mention food at all, but only, for example, encourage increased physical activity by children. However, such advertisements are usually still heavily identified with the company's logo and visual symbols. There may be room for debate and confusion as to whether they are advertisements for food, strictly speaking, but there is no reason to doubt that they would have the effect of increasing children's favourable disposition towards the company's brand, and therefore also their preference for its food.

Arguably, then, an effective system for minimising the impact of advertising on children's food preferences and nutrition behaviours should be addressed at brands as well as at particular products. While on the surface this could look like victimisation of the companies in question, or some form of "guilt by association", it would have the virtue of addressing the way that advertising works, by establishing brand loyalty. We are not aware of any regulation, anywhere in the world, using such an axis.

Rather, there are numerous lesser restrictions, short of a ban, that apply to all food advertising. For example, in Australia Children's Television Standard (CTS) 19(6) states:

An advertisement for a food product may not contain any misleading or incorrect information about the nutritional value of that product.

Other provisions, such as Clause 6.23 of the Commercial Television Industry Code of Practice, and clauses (b)7, (b)8 and (b)9 of the Children's Advertising Review Unit (CARU) Self-Regulatory Program for Children's Advertising in the United States, single out food advertisements for special scrutiny. Clause 6.23 provides:

6.23 Advertisements directed to children for food and/or beverages:

6.23.1 should not encourage or [expressly endorse] [not engaging in any or much physical activity as a way of life];

6.23.2 should not encourage or [expressly endorse] [excessive or compulsive consumption of food and/or beverages] ....

The Australian Association of National Advertisers (AANA) Advertiser Code for Advertising to Children contains a similar provision in Clause 2.10, except that to fall foul of it, an advertisement must encourage or promote *both *inactive lifestyle *and *overconsumption.

The US provisions state as follows:

7. The amount of product featured should not be excessive or more than would be reasonable to acquire, use or consume by a person in the situation depicted. For example, if an advertisement depicts food being consumed by a person in the advertisement, or suggests that the food will be consumed, the quantity of food shown should not exceed the labelled serving size on the Nutrition Facts panel; where no such serving size is applicable, the quantity of food shown should not exceed a single serving size that would be appropriate for consumption by a person of the age depicted.

8. Advertising of food products should encourage responsible use of the product with a view toward healthy development of the child. For example, advertising of food products should not discourage or disparage healthy lifestyle choices or the consumption of fruits or vegetables, or other foods recommended for increased consumption by current USDA Dietary Guidelines for Americans and My Pyramid, as applicable to children under 12.

9. Advertisements for food products should clearly depict or describe the appropriate role of the product within the framework of the eating occasion depicted.

a. Advertisements representing a mealtime should depict the food product within the framework of a nutritionally balanced meal.

b. Snack foods should be clearly depicted as such, and not as substitutes for meals.

The AANA has propounded an entire code dedicated to food and beverage advertising, the Food and Beverage Advertising and Marketing Communications Code.

The extent to which food advertisements might fall foul of these provisions depends on the application of criteria that go beyond the mere fact of the advertisement being a food advertisement. However, the regulations set out above provide illustrations of the kinds of restrictions that could turn on the "food" axis. The very variety of approaches available makes it difficult to comment on the food axis in a general way, other than to say that the presence in numerous instruments of regulations revolving on this axis suggests a wide recognition of the sensitivity of food as a category.

#### "Junk" food

There is a compelling logic to the idea of limiting advertising restrictions to foods considered to be especially likely to contribute to obesity in children. Most people would instinctively include on any such list "fast" foods such as hamburgers, chips and fried chicken; confectionery including chocolate; ice cream; and salty snacks such as potato chips. However, drawing the boundaries between healthy and unhealthy foods is not a simple matter as it must always involve balancing the nutritional value of the food with any excessive levels of fat or sugar.

For example, flavoured yoghurt contains the beneficial nutrients of protein, calcium and vitamins – but also a considerable amount of sugar. It is a healthier snack than, say, chocolate, but overconsumption can still contribute to obesity. Reasonable minds will disagree on whether it should be included or excluded from a list of foods to be subject to advertising restrictions in the name of preventing childhood obesity.

There are various ways of dividing food up into "good" and "bad" categories for the purposes of regulation. One is nutrient profiling, which is the approach that the UK has recently adopted in order to identify foods high in fat, sugar and salt – referred to as HFSS foods. The Australian Communications and Media Authority Issues Paper for the Children's Television Standards Review said that "this option [is] currently unviable in the Australian context" but did not explain why the British system could not be adopted here. Nor did it appear to recognise the existence under Food Standards Australia and New Zealand of a profiling system [12].

Another possibility in Australia would be to use the distinctions between basic food items and luxuries that have been drawn up for the purposes of the goods and services tax [13]. This would have the virtue of a degree of familiarity within the Australian community; but on the other hand it would not necessarily distinguish neatly and accurately between healthy and non-healthy foods.

Regulations revolving on the "junk food" axis share the problem adverted to earlier, of advertisements for brands rather than particular products. Indeed the problems are more deeply ingrained in the case of a "junk food" axis because restaurant chains that have traditionally specialised in burgers and fried foods have recently started to introduce healthier alternatives. The same would be said of a number of food and beverage companies that produce a range of products, some of which are healthier than others. A regulatory system that was serious about limiting the effect of advertising on children's food preferences and nutrition behaviours would include restrictions on the use of more general advertising that could attract children to a brand, or an outlet, where unhealthy food dominates.

#### Children's food

In some cultures, it is possible at some level to distinguish between children's foods and other foods, and there is a superficial logic to singling such foods out for special attention when imposing restrictions on advertising for the protection of children.

An example of such an approach is in the Australian Association of National Advertisers Advertiser Code for Advertising to Children (ACAC). As we shall see in the next section, the ACAC applies only to advertising that is aimed at children; its application is limited further in that the product being advertised must be one which is "targeted toward" children and has "principle [sic] appeal" to children (see Clause 1(c) in conjunction with (b)). Therefore, in so far as the Code applies to food advertising, it applies only to this subset of foods.

As with the notion of children's programming, children's food is not an easy category to define. There is considerable room for debate as to whether products like hamburgers, fried foods, chocolate and potato chips would be seen as being in the category, or whether their appeal to adults would rule them out. Yet these are clearly some of the foods for which children have been developing too great a preference and effective anti-obesity regulation should include them.

Moreover, the very vagueness of the category would make it difficult to know whether it would be worth complaining against an advertisement for some major categories of food.

### 3. The (apparent) target audience

The majority of regulatory rules for the protection of children from advertising single out for added restrictions advertising that has children as its target audience. For example, Quebec and Sweden are well-known for having strict rules to protect children from advertising; this is the kind of axis used in both places. In Quebec, s 248 of the *Consumer Protection Act 1980 *provides in part:

no person may make use of commercial advertising *directed at *persons under thirteen years of age. (emphasis added)

It is worth noting that this provision extends to all advertising, not just food and not just that on television. The Swedish ban appearing in Chapter 7, s 4 of the *Radio and Television Act 1996*, by contrast, applies only to television:

Commercial advertising in a television broadcast *may not be designed to attract the attention of *children under 12 years of age. (emphasis added)

Australian regulations contain provisions that divide the world up in a similar way. For example, Clause 6.20 of the Commercial Television Industry Code of Practice applies the advertising restrictions from the Children's Television Standards to advertising "directed to children" and Clause 1(c) of the Advertiser Code for Advertising to Children applies to "Advertisements which ... are directed primarily to Children". (As mentioned above, this latter provision is further restricted to advertisements for children's products).

The first question about such an axis is whether the judgment is subjective or objective: does the regulation cover advertisements whose authors *intend *to catch children's attention, or advertisements that are expected to have that effect irrespective of intent? The Swedish provision, in particular, makes it sound as if it might be the former, which is clearly the narrower of the two approaches. However, the more usual approach is a broader one of providing criteria for determining whether an advertisement meets the definition. Section 249 of the Quebec legislation provides:

To determine whether or not an advertisement is directed at persons under thirteen years of age, account must be taken of the context of its presentation, and in particular of:

(a) the nature and intended purpose of the goods advertised;

(b) the manner of presenting such advertisement;

(c) the time and place it is shown.

In Sweden, the three criteria used for assessing whether an advertisement is "designed to attract the attention of children under 12 years of age" are roughly the same: the design of the advertisement, the type of product and the context of the broadcast [14]. In the USA, the *Children's Advertising Review Unit Self-Regulatory Program* document lists four factors, revolving around the adjacent programming and the apparent intent of the advertiser (see paragraph II(A)(1)(a)-(d)).

In Australia, the official publication containing the Commercial Television Industry Code of Practice (CTICP) contains a document entitled *Advisory Note: Commercials or Community Service Announcements Directed to Children*, which is stated to be:

intended to provide guidance on the factors licensees will consider in assessing *who *[sic] *a commercial is directed to *for the purpose of applying Clause 6.23 of the Code *"Commercials or Community Service Announcements Directed to Children"*. (emphasis in original)

Clause 6.23, set out above, is a provision relating specifically to food advertisements. The factors for consideration under the Advisory Note are:

• the nature of the product or service, and the persons most likely to be interested in that product or service – is the product or service one for which children are the only users or form a substantial part of the market?;

• the theme of the commercial – are adult or children's themes used? For example, characters such as monsters, animals and the like;

• the 'story line' and the approach taken in selling the product or service – is the story line aimed at children? For example, does the commercial have a simple uncomplicated plot structure such as 'good' against 'evil'?;

• the visuals used in the commercial – are the visuals aimed at children? For example, the commercial uses animation or imaginative visuals which appeal to children;

• the language of the commercial – does the commercial use children's language?;

• the age of actors appearing in the commercial – are child actors depicted actively using a product or service for which children constitute the market?; and

• the target audience for the commercial – is the target audience children? This is relevant where the other factors set out above indicate that a commercial is intended to appeal to children.

It remains to be seen just how these factors will work in practice. For example, is it sufficient for one criterion to be met, or must they all be met before an advertisement will be thought of as "directed at children"?

It may be useful to consider the example of an advertisement that the broadcaster did not consider to be "directed to children", albeit under the previous version of the Code which did not contain the Advisory Note. This was one where an adult addressed comments (ostensibly) to parents about the vitamins and minerals in a highly sugared breakfast cereal. The advertisement was considered not to be directed to children [15] even though (a) the product is one that is of primary interest to children, and (b) the adult concerned is a well-known children's entertainer.

Under the Advisory Note, the first factor would suggest that the advertisement was directed to children, but the rest would suggest the opposite. Therefore, if a balancing approach were taken, we might expect that the result would be the same: the advertisement would not be considered to be "directed to children". It is interesting to note that the presenter's status as a children's entertainer is not "caught" by any of the factors. She is not a child actor depicted using the product, but neither is she unrecognisable to children.

Another example of how this kind of axis works in practice comes from Quebec: in an advertisement for a fast-food restaurant chain, a man is shown taking a young boy to the restaurant. The child is clearly enthusiastic about being there, and is shown at the table with a child's meal in front of him. But the "story line" of the advertisement is the man's interest in the number of attractive women in the restaurant eating salads. He is shown noticing them, then at the end of the advertisement the boy says, "There are lots of ladies here, aren't there?" and the man says, "Really? I hadn't noticed." Therefore on the surface, the advertisement is addressed to single heterosexual men. Also it places greater emphasis on the restaurant chain's salad lines than on the unhealthy fast-food for which it is traditionally known. Unless a single element is sufficient to satisfy the Advisory Note to Clause 6.23 of the CTICP (which seems unlikely), this advertisement would pass muster under the CTICP as not being "directed to children". Although the little boy is seen 'actively using a product or service for which children constitute the market', every other element of the advertisement is contrary to what is described in the Advisory Note. The advertiser would claim that the 'product or service' being advertised is the salads, not one 'for which children are the only users or form a substantial part of the market'. The theme of picking up attractive women is not a children's theme, and for the same reason the 'story line' is clearly not aimed at children. The visuals are not child-oriented, in the sense that there is no animation or similar, and the language is not children's language but rather a life-like conversation between an adult and a child. The advertisement was presumably permissible under Quebec's strict laws for much the same reasons, under the "manner of presenting" consideration contained in s 249(b). Yet there is nothing to suggest that a child would not have noticed the advertisement, and received its selling message about the chain.

A third example comes from Swedish television. An advertisement for cheesy snacks shows a cartoon-style mouse superhero ("Mouseman") rescuing a cartoon-style old lady (conservative 50 s-style skirt suit and pillbox hat with a veil) who has been mugged by some cartoon-style robbers (striped jumpsuits and eye masks), to deprive her of a large piece of cartoon-style cheese she is carrying (a wedge, with holes). However the advertisement is not a cartoon, rather it is live action with a cartoon-like design. Also, it is in English. These, presumably, were the reasons it was able to be shown on Swedish television. Yet once again there is no reason to think that a Swedish child's attention would not be drawn by the advertisement. Still less could one say that a child seeing the advertisement would not have been exposed to food advertising.

In all of the above systems, consumers and regulators are provided only with a list of factors to take into consideration, and not with a definition as such. The need to balance a number of different considerations means that it is impossible to say with certainty what the conclusion will be, and this is telling in itself. In any system which, like Australia, relies on complaints from consumers to alert authorities to possible breaches, the number of complaints is bound to be minimised by maximising uncertainty as to whether a breach has occurred.

The foregoing discussion shows that regulatory axes of this kind – that is, those that centre on some notion of a target audience for the advertisement – have a superficial appeal but are inherently vague and open to interpretation. This is especially so where multiple factors need to be balanced against each other. It would be far easier for consumers to identify breaches under a "single factor" test, where heightened scrutiny was activated by any one factor indicating children are targeted.

In addition, target audience tests have a limited capacity to restrict children's exposure to food advertising, because there is no reason to think that they do not notice any other kind of advertisement. The research on the impact of food advertising on children's choices has not been limited to the impact of children's advertising in this sense, so there is no reason to limit regulations in this way either. We argue that a regulation applying only to advertising aimed at children is inherently inappropriate and ill-adapted to addressing the contribution of food advertising to childhood obesity.

### 4. The time of day

The discussion above suggests that it is better, and easier for consumers, if regulation uses different times of day, rather than the type of program, the type of product or the target audience, for determining the level of advertising restriction that applies. Reliance on times of day has the important virtue of centring levels of regulation on an objectively verifiable fact, and it is not left to consumers or regulators to interpret more subjective criteria. Such certainty may also be beneficial for broadcasters.

The current regulatory system in Australia does not draw lines according to times of day, except in the general sense that the Commercial Television Industry Code of Practice (CTICP) lays down classification zones during which material must meet certain criteria relating to matters such as coarse language, sexual references and adult themes. While these restrictions extend to advertisements, they do not have any particular application to food advertising.

#### When children are watching

There are three possible ways of dividing up the day and the week according to when children are watching. One is to make observations about how children spend their day and when they will therefore be available for television watching. The presumption is then made that many children *will *be watching at those times. This happens to some extent in the setting of classification zones on Australian television: for example, children are presumed to be in school until 3.30 pm on weekdays, so under Clause 2.8 of the CTICP, the "G" classification zone does not start until 4.00 pm. It is otherwise during school holidays: see Clause 2.9. This might be called the 'opportunity to watch' approach.

The other two ways of setting time zones are based on consideration of data on actual viewing patterns, but use them in different ways. One looks at when children make up a given proportion of the audience; the other looks at the proportion of children who would be expected to be watching at a given time. Both of these are based not on opportunity but on actual audience information.

The first of these two approaches is more favourable to privileging the commercial interests of the TV station which would wish to have access to the greatest number of adult viewers. If children are a large proportion of the audience, that means there are relatively few adult viewers to whom access is being restricted. The more adult viewers, the more the broadcaster has to lose as a result of advertising restrictions. However, such an approach has only limited capacity to restrict children's exposure to food advertising. The fact that a large number of adults might be watching does not change the potential impact of the advertising on the child audience.

A more effective approach is to consider what proportion of children are expected to be in the television audience at a given time. If restrictions are tighter at times when large numbers of children are watching, this means that more children are having their exposure limited.

Whichever approach is taken, it is important to nominate the times of day for different levels of restriction in advance, and for these zones to be reasonably stable. It would not assist the effectiveness of the scheme if the zones shifted from one week to the next, or even one month to the next. Consumers should be able to ascertain with ease which zone they are in, so as to determine what level of restriction applies. Therefore it would be necessary to make some generalised predictions about the make-up of audiences, rather than trying to micro-manage the audiences for particular programmes in particular weeks.

This means that "when children are watching" criteria cannot be expected to tie regulation, all the time, to factually accurate information about audiences. There is always scope for variations (up or down) when particular shows are screened. On the other hand, programming does tend to follow viewing patterns, so times of day would normally be a reasonable proxy for the size of the child audience.

#### A watershed

Using a time of day as a watershed for regulating food advertising for the protection of children has the benefit of simplicity. If the watershed were, say, 9 pm, the rule would be: no food advertising before 9 pm. A watershed could also be applied in a more nuanced way; for example, no ads for foods high in fat, sugar or salt before the watershed, no use of premium offers before the watershed, and so on. This would have the very substantial benefits of simplicity and certainty, though of course at the cost of sacrificing a degree of finesse and accuracy in targeting times and programs when children are actually, or even likely to be, watching. Therefore, a simple watershed rule would risk overstepping the mark, by biting into broadcasters' profits without any necessary corresponding benefit to children.

### 5. The content of the advertisement

Space does not permit a detailed examination of regulatory axes centring on the advertisement itself. In this section, however, we provide a few indications of what such axes consider to be relevant features, and the factors that limit the effectiveness of some such axes. In addition to the matters discussed below, it is worth noting that some of the elements used to determine whether an advertisement is aimed at children also refer to elements of content (see eg Cl 6.23 of the CTICP, quoted above).

#### Use of personalities

Many regimes have specific rules about the use of personalities in children's advertising, for example Children's Television Standard 22 (Australia); *Radio and Television Act 1996*, Chapter 7, Section 4 (Sweden); Broadcast Code of Advertising Practice, section 7.2.4 (UK); *Code for Advertising to Children*, Guideline 2(l) (NZ). The recent popularity of this advertising strategy suggests that it is seen by industry as having a significant impact on children, but on the other hand the fact that such advertisements continue to be broadcast suggests that the rules are not very effective in curtailing it. This may be because they are not always sufficiently broad to catch the full range of personalities (for example, sporting heroes) that might appeal to children.

#### Premiums

Another popular way of promoting products to children is to offer some kind of give-away, or premium. Many systems contain rules limiting the presentation of premiums in the context of advertising. However, in Australia at least, these have been interpreted so as to allow the marrying of food and non-food items in a single "product" so that a toy given away with food is not considered to be a "premium" and is therefore not caught by the restrictions [16].

#### Pester power

"Pester power" is a term that refers to the ability of children in many families to gain access to the products they desire by wearing their parents down so that they give in and purchase. Its documented efficacy [17] suggests it would be a powerful link in the chain of causation between food advertising and obesity.

Many regulatory provisions appear to address pester power by making some reference to "undue pressure". For example, Children's Television Standard 18 provides that "A licensee may not broadcast any advertisement designed to put undue pressure on children to ask their parents or other people to purchase an advertised product or service." However the "undue pressure" this provision addresses is pressure from the advertisement, on children. Pester power is at work when children place undue pressure on parents. Therefore CTS 18 and provisions like it do not really limit the role of pester power in providing a link between food advertising and childhood obesity.

Moreover, so-called anti-pester power provisions tend to assume that the reason children pester their parents is that an ad has instructed them to do so. This is not the case, but rather children pester when they want a product badly enough. Realistically, then, the only way to do away with pester power is to do away with effective advertising to children.

#### Misleading or deceptive

Consumer protection legislation against misleading and deceptive conduct in the promotion of goods and services is both well-known and ubiquitous [18]. In the context of food advertising, it is not sufficient to say that misleading information is disallowed; it is necessary to address the overall picture being painted of an unhealthy product. For example, a sugary breakfast cereal might be rich in calcium, and lollies are typically fat-free. Many provisions allow advertisers to focus on these positive aspects without making reference to others that make the product, overall, an unhealthy one. To be effective, advertising regulation needs to disallow selective reference to minor nutritional attributes.

#### Promoting unhealthy lifestyles

Recent reviews of industry codes have seen the emergence of provisions disallowing the encouragement of unhealthy practices such as overeating or inactive lifestyles [19,20]. There is a serious question as to whether such provisions prevent the broadcast of any advertisement that any advertiser would otherwise want to have shown. Advertisers over the years have shown themselves much more interested in associating their unhealthy food products with health and physical vigour.

Therefore, it is difficult to escape the conclusion that such provisions in advertising codes have little or no effect on the exposure of children to food advertising. There is simply no advertisement that they would otherwise see, that is made unavailable by these provisions.

## Conclusion

This article has described a number of axes around which regulation revolves. Each axis represents a specific criterion that singles out for stricter regulation certain advertising to children, including food advertising. We have seen that some of the criteria commonly used rely on vague or subjective notions that make it difficult to determine in advance whether a breach has occurred. This has two effects: it makes it less likely that a consumer will take the trouble to complain, and it makes it harder to obtain a breach finding. The more serious the misconduct that the regulation seeks to capture, the more likely that regulators and decision-makers will be reluctant to adopt an interpretation that makes an advertiser "guilty" of that misconduct.

In addition, not all criteria in use have a capacity to moderate the impact of television food advertising on children's food preferences, or their diets. For example regulations tend to assume that children are more influenced by advertising that is in some sense aimed at them. Not only is this an inherently vague category, but the assumption is not supported by the research [6].

The most effective means of moderating the impact of food advertising on children's food preferences is to limit their exposure to it, and the regulatory criterion with the greatest capacity to do so is one based on the time of day when a certain number of children are (expected to be) in the audience.

## Competing interests

The authors declare that they have no competing interests.

## Authors' contributions

CN did the initial research on the content of the various regulatory regimes; all authors developed the ideas and analysis through discussion amongst them. All have been involved in the preparation of a major report on the outcomes of the study. EH drafted this article and all authors read and approved the final manuscript. JC revised the article following suggestions from the editors.
